# Valorization of Avocado Seed Wastes for Antioxidant Phenolics and Carbohydrates Recovery Using Deep Eutectic Solvents (DES)

**DOI:** 10.3390/antiox12061156

**Published:** 2023-05-26

**Authors:** Alexandra Del-Castillo-Llamosas, Fernando Rodríguez-Rebelo, Beatriz Rodríguez-Martínez, Adrián Mallo-Fraga, Pablo G. Del-Río, Beatriz Gullón

**Affiliations:** 1 Departamento de Enxeñaría Química, Facultade de Ciencias, Universidade de Vigo, 32004 Ourense, Spain; alexandra.castillo@uvigo.es (A.D.-C.-L.); frodriguez@uvigo.es (F.R.-R.); beatriz.rodriguez@uvigo.es (B.R.-M.);; 2 Stokes Laboratories, School of Engineering, Bernal Institute, University of Limerick, V94 T9PX Limerick, Ireland

**Keywords:** avocado seed, antioxidant phenolics, biorefinery, deep eutectic solvents, microwave, enzymatic hydrolysis

## Abstract

Avocado seeds represent the chief waste produced in avocado processing, leading not only to environmental problems regarding its elimination but to a loss of economic profitability. In fact, avocado seeds are known as interesting sources of bioactive compounds and carbohydrates, so their utilization may reduce the negative effect produced during the industrial manufacture of avocado-related products. In this sense, deep eutectic solvents (DES) are a novel greener alternative than organic solvents to extract bioactive polyphenols and carbohydrates. The study was based on a Box–Behnken experimental design to study the effect of three factors, temperature (40, 50, 60 °C), time (60, 120, 180 min) and water content (10, 30, 50% *v*/*v*) on the responses of total phenolic (TPC) and flavonoid content (TFC), antioxidant capacity (measured as ABTS and FRAP) and xylose content in the extract. The DES Choline chloride:glycerol (1:1) was used as solvent on avocado seed. Under optimal conditions, TPC: 19.71 mg GAE/g, TFC: 33.41 mg RE/g, ABTS: 20.91 mg TE/g, FRAP: 15.59 mg TE/g and xylose: 5.47 g/L were obtained. The tentative identification of eight phenolic compounds was assayed via HPLC-ESI. The carbohydrate content of the solid residue was also evaluated, and that solid was subjected to two different processing (delignification with DES and microwave-assisted autohydrolysis) to increase the glucan susceptibility to enzymes, and was also assayed reaching almost quantitative glucose yields. These results, added to the non-toxic, eco-friendly, and economic nature of DES, demonstrate that these solvents are an efficient alternative to organic solvents to recover phenolics and carbohydrates from food wastes.

## 1. Introduction

Nowadays, fruit and vegetable consumption entail the generation of a wide amount of waste with no apparent value that is sometimes thrown into landfills or burned for energy production [1,2]. The generation of these wastes occurs in industrial processing of fruit and vegetables to manufacture goods such as juices, jams, dehydrated fruit, etc. [2]. Owing to its elevated moisture and microbial load, the residues from this type of food are highly polluting [3]. In this sense, the valorization of these wastes to obtain value-added products can lead to a financial and environmental improvement owing to the development towards a circular economy and in accordance with the zero waste policies proposed by the 2030 agenda [1,4,5,6].

In the specific case of avocado, its production, exportation, and consumption have been incremented in recent years [7,8]. One of the main productive countries (Mexico), [6] reached 2.5 million tons of this fruit in 2021, 35% higher than ten years prior [9]. Focusing on Europe, Spain is the largest avocado producer (117,000 tons in 2021), representing almost 75% of the European production of this fruit [9]. The global avocado exportation increased 8.1% from 2019 to 2020, corresponding to 2.3 million tons [10]. This increase is related to its consumption, which is expected to keep rising as this product can be consumed not only fresh but also as processed product such as oil, guacamole, and many other pulp-derived products [11,12].

In this sense, the avocado industry must deal with a high amount of waste disposal, since it generates massive quantities of by-products (seed and peel) which represent between 21–30% of the raw material [13,14]. These by-products are proteins (3–9 wt%), carbohydrates (43–85 wt%), lipids (2–9 wt%), minerals (2–6 wt%), and poly-phenols [4,6,12,15]. Due to its interesting composition, its use may be of interest to produce highly value-added compounds. Phenolic compounds possess well-known biological activities such as antibacterial, anti-inflammatory, antimicrobial, anticarcinogenic, chemopreventing, or epigenetic effect between others [1,16,17].

One way to recover bioactive molecules from fruit by-products is by using deep eutectic solvents (DES) [18,19]. DES are conformed by a blend of a hydrogen-bond acceptor (HBA) and a hydrogen-bond donor (HBD) in a suitable molar ratio that allows to form the eutectic mixture. The HBA most widely used in the literature is choline chloride, a quaternary ammonium salt with interesting properties, being non-toxic, cheap, and biodegradable. On the other hand, the typically employed HBDs are organic acids, polyols, sugars and urea, among others [18,20,21]. These DES are widely used for extracting polyphenolic compounds [20], improving the extraction compared with commonly organic solvents [16,20], as they are rapidly prepared, easy to be stored, non-flammable, inexpensive, and present a high capacity of extraction for both polar and non-polar compounds [19,21]. There are different studies with some examples regarding the extraction of bioactive compounds from fruits using DES such as berry by-products [22], olive pomace [23], or apple pomace [24].

The extraction of bioactive compounds with DES does not really affect the structure of avocado seed since it is a mild pretreatment, so the use of a subsequent treatment would allow a greater enzymatic digestibility of the remaining glucan. Among them, two strategies were evaluated in this work: delignification with DES and microwave-assisted autohydrolysis. The first one represents an interesting way for using DES not only for the extraction of phenolics at milder conditions, but for the solubilization of lignin at harsher conditions [25]. Conversely, microwave irradiation, in the presence of water, is a promising method since it helps in biomass fractionation, which improves enzymatic hydrolysis and therefore sugar yield [26,27,28]. Among the advantages of using microwaves are heating occurs directly on the biomass with uniform distribution and can be stopped immediately, its ecological character, the degradation of hydrogen bonds due to electro-magnetic radiation, the low consumption of solvents, energy saving, etc. [26,29].

To the best of our knowledge, the use of DES for to recover bioactive compounds from avocado seeds has not been reported in the bibliography, thus, that is the key aim of this study. For that reason, the extraction of bioactive compounds from avocado seed was optimized. The water content in the DES, the temperature, and the residence time were studied and the total phenolic content (TPC), total flavonoid content (TFC), the antioxidant capacity (ABTS and FRAP) and xylose content in the extract were evaluated. The identification of phytochemicals using HPLC-ESI was developed. The spent solid was subjected to two different processing in order to increase and assay the enzymatic susceptibility of the remaining glucan, providing a comprehensive usage of the avocado seed waste.

## 2. Materials and Methods

### 2.1. Materials

Avocado seed (AS), from the Hass variety, was gathered from a local restaurant in Ourense (Northwest Spain). Firstly, the seed was washed with tap water with the purpose of removing the remaining pulp and the layer covering the seed was removed. Before drying it at room temperature, AS was powdered using a laboratory mill with sieve to a smaller size than 1 mm. AS was collected into zip bags and stored at −20 °C.

The chemicals used in this work were 2,2′-azino-di(3-ethyl- benzo-thia-zoline-6-sulfonic acid (ABTS), 2,4,6-tri(2-pyridyl)-s-triazine (TPTZ), 6-hydroxy-2,5,7,8-etra-methylcrhoman-2-carboxylic acid (Trolox), arabinose, choline chloride, ethanol, Folin–Ciocalteu reagent, furfural, gallic acid, glucose, glycerol, hydrochloric acid, hydroxymethylfurfural, iron (III) chloride hexahydrate, methanol, potassium persulfate, rutin, sodium acetate trihydrate, sodium carbonate, sulfuric acid and xylose, that were purchased from Sigma-Aldrich (Barcelona, Spain).

### 2.2. Chemical Characterization of the Feedstock

AS was characterized by NREL methods for moisture [30], ash [31], and ethanol extractives [32]. The extractives-free AS was subjected to quantitative acid hydrolysis [33] for polysaccharides determination in triplicate. Briefly, 0.2 g of AS were mixed thoroughly with 2 mL of sulfuric acid (72% *w*/*w*) during 1 h at 30 °C. Afterwards, the sulfuric acid concentration was diminished to 4% with deionized water and placed in an autoclave at 121 °C for 1 h. The resulting blend was filtered to obtain (i) a solid, known as Klason lignin, which was placed in an oven for 24 h at 105 °C before quantification, and (ii) a liquid fraction that was filtrated via 0.45 μm nylon membranes and injected in HPLC (Agilent 1200 series, Palo Alto, CA, USA) for monosaccharides and organic acids determination. The conditions employed for the chromatography were: 5 µL of sample injected in a Rezex ROA Organic Acid H+ (Phenomenex) at 60 °C, refractive index detector at 35 °C, mobile phase of 3 mM sulfuric at a flow of 0.6 mL/min [34]. The determinations were performed in three replicates.

The uronic acids were assayed by the method stated by Blumenkrantz and Asboe-Hansen [35] on the liquid fraction after quantitative acid hydrolysis. The evaluation was carried out in three replicates.

### 2.3. Experimental Design for Extraction Using Deep Eutectic Solvents (DES)

Choline chloride:glycerol (1:1) DES was synthetized by mixing the appropriate molar proportions with the necessary water (between 10–50% *v*/*v*) with magnetic agitation at a temperature lower than 80 °C until becoming transparent.

DES were combined with AS at a solvent–solid ratio of 15 mL/g and the mixture was introduced in an orbital shaker for different residence times (60–180 min) and temperatures (40–60 °C). Additionally, 50% *v*/*v* and 80% *v*/*v* ethanol were employed as control solvents due to their capacity as widely used organic solvents to extract antioxidant phenolics.

The effect of temperature, time, and water content was evaluated by response surface methodology (RSM) using a Box–Behnken design with a triplicate in the central point, and fitted to a second-order polynomial:

$$y_{j}=\beta_{0}+\overset{3}{\underset{i=1}{\sum }}\beta_{i}x_{i}+\sum \overset{3}{\underset{i<j=1}{\sum }}\beta_{ij}x_{i}x_{j}+\overset{3}{\underset{i=1}{\sum }}\beta_{ii}x_{i}^{2}$$

where y_j_ represents the dependent variables (j = 1–5), β_0_, β_i_, β_ij_, and β_ii_ represent the regression coefficients determined from the experimental data via the least-squares methodology, and x_i_ and x_j_ represent the independent variables (dimensionless and normalized), that can vary between −1 and 1. Microsoft Excel’s Data Analysis Add-In, USA, was employed to fit the experimental results employing a regression analysis. The suitability of the model was evaluated by assessing the absence of fit, the coefficient of determination (R^2^), and the F-test value determined by the analysis of variance.

In order to obtain the maximum values of the response variables simultaneously, a multi-response surface optimization was employed. Optimized conditions were assessed with the software STATGRAPHICS Centurion XVI (version 16.1.11). Model validation was performed by carrying out the experiments at the optimum conditions of extraction and comparing the predicted and experimental values.

### 2.4. Chemical Characterization of Liquid and Solid Fractions after the Extraction

Part of the liquid phase was directly filtrated through 0.45 μm nylon membrane and injected into HPLC to quantify monomeric sugars (glucose, xylose, arabinose), acetic acid, hydroxymethylfurfural (HMF) and furfural (F), using the methodology stated before (Section 2.2).

The solid fraction was characterized by quantitative acid hydrolysis as described in Section 2.2.

### 2.5. Determination of Total Phenolic Content (TPC) and Total Flavonoid Content (TFC)

The following assays were carried out to quantify the phenolic and flavonoid content from the liquid fractions after DES extraction. For TPC, the Folin–Ciocalteau method as explained by Singleton et al. [36] was employed, expressing the results in mg of gallic acid equivalents (GAE)/g AS. For the case of TFC, the aluminum chloride method described by Blasa et al. [37] was employed, expressing the results in mg of rutin equivalents (RE)/g AS. The determinations were carried out in three replicates.

### 2.6. Antioxdant Capacity Assays

The liquid fraction after DES extraction was subjected to two antioxidant capacity assays: 2,2-azino-bis-3-ethylbenzothiazoline-6-sulphonic acid (ABTS) radical cation decolorization evaluation and ferric reducing antioxidant power (FRAP) as explained by Gullón et al. [38]. The data were measured in mg of Trolox equivalents (TE)/g AS. The determinations were performed in three replicates.

### 2.7. Identification and Quantification of Phytochemicals Using HPLC-ESI

The liquid fraction from DES extraction at optimized conditions was extracted with ethyl acetate (ratio 1:1 *v*/*v*). Both the extract and ethyl acetate were magnetically stirred at room temperature for 15 min and let separate in a decanting ampoule, recovering the ethyl acetate and repeating the extraction on the DES extract two more times at the same conditions. The recovered ethyl acetate was then rotatory-evaporated at 40 °C until the solvent was completely removed, and the resulting sample was resuspended in methanol and injected in HPLC (Agilent 1260 series, Palo Alto, CA, USA) with a AB SCIEX Triple Quad 3500 detector (AB Sciex, Foster City, CA, USA) and equipped with an electrospray source of ionization (ESI). An amount of 5 µL of filtered (0.45 µm membranes) sample was injected in a Luna C18 column (Phenomenex), using as mobile phase A formic acid 0.1% and as mobile phase B acetonitrile with formic acid 0.1%, at a flow rate of 0.3 mL/min. The ionization source was positive and negative with turbo V™, employing nitrogen as nebulizer and collision gas, and the data acquisition was performed by means of a multiple reaction monitoring (MRM) considering the mass of the parent ion and its transitions.

### 2.8. Processing of the Phenolic-Free Avocado Seed to Increase Enzymatic Digestibility: DES-Delignification or Microwave-Asssisted Autohydrolysis

To increase the digestibility of the spent solid after the extraction of antioxidant phenolics with DES, two pretreatment strategies were followed: a delignification with DES and a microwave-assisted treatment with water.

The first strategy consisted of a delignification using the same DES previously employed for the phenolics extraction. The solid and DES were mixed at a LSR of 15 mL/g and introduced in an autoclave at 121 °C for 60 min. These conditions were selected according to a previous study (data not shown). After the pretreatment, the solid and the liquid phase were filtered and the solid was collected, washed with ethanol 50% (*v*/*v*) at 50 °C and water, and dried at room temperature.

Alternatively, microwave-assisted autohydrolysis was performed in a Monowave 450 single-mode microwave reactor (Anton Paar GmbH) rigged with an air compressor in order to lessen the temperature of the experiment. The solid and water were mixed within a LSR of 15 mL/g, heated as fast as possible to achieve the target temperature (200 °C) and maintained for 5 min. Temperature was measured by the IR detector, stirring speed was set at 850 rpm, and the cooling time was 5 min. These conditions were selected according to a previous study. The reactions were carried out in standard Pyrex vessel of 30 mL volume (G30). The liquor was collected after filtration.

### 2.9. Enzymatic Susceptiblity of the Spent Solid

Considering the chemical composition of AS, rich in glucan, the solid obtained after the DES delignification and the liquid obtained in the microwave-assisted autohydrolysis were assayed as a substrate to manufacture fermentable sugars. For the evaluation of the susceptibility of the solid and liquid, enzymatic saccharification assays were performed under the optimum conditions of the enzymes (Celluclast 1.5 L cellulases and 188 Novozyme β-glucosidase, provided by Novozymes). The cellulase activity of Celluclast was evaluated by filter paper assay [39] and the value obtained was 116 FPU/mL and, for β-glucosidase, the activity was measured by means of international units (IU) achieved a value of 2337 UI/mL for 188 Novozyme [40]. The conditions set in the enzymatic hydrolysis of both solid after delignification and liquor after microwave-assisted autohydrolysis were an enzyme–substrate ratio (ESR) of 25 FPU/g, and a β-glucosidase-to-cellulase ratio of 25 IU/FPU. In the solid experience carried out, the liquid–solid ratio (LSR) was set at 20 g/g. Enzymatic hydrolysis were carried out at conditions of 50 °C and pH 4.85 (for which a 0.05 N citric acid buffer was used) with an orbital agitation of 150 rpm. Samples of both enzymatic hydrolysis were withdrawn between times 0–48 h. The supernatant was obtained by centrifugation to subsequently filter it through a 0.45 µm membrane to proceed to the HPLC analysis for monosaccharides quantification. To obtain the glucose yield of the solid, the potential glucose was calculated according to the formula:

$$Gpot=Gn\cdot \frac{180}{162}\cdot \frac{\rho }{LSR+1-KL}$$

where Gn represents the glucan content per 100 g of AS solid, 180/162 denotes the stoichiometric factor for glucan hydration upon hydrolysis, ρ corresponds to the density of the reaction medium (an average value of 1005 g/L), LSR represents the liquid-to-solid ratio, and KL is the Klason lignin content per 100 g of pretreated AS.

On the other hand, to obtain the potential glucose of the liquid phase from the microwave treatment with water, a quantitative acid posthydrolysis (4% H_2_SO_4_, 121 °C, 20 min) was performed to obtain the maximum glucose content taking into account monosaccharides and oligosaccharides of glucose.

### 2.10. Statiscal Analysis

Results from TPC, TFC, ABTS, FRAP, and xylose for the solvent selection (quantified as mean value ± standard deviation), were underwent to statistical analysis via the software R (version 4.1.0). A one-way ANOVA (analysis of variance) followed by a Tukey’s test to determine the statistical differences among samples, considered significant when *p* < 0.05.

## 3. Results

### 3.1. Feedstock Characterization

The chemical composition of the avocado seed is as follows (expressed as g of component/100 g of AS in dry weight ± standard deviation): glucan 54.73 ± 0.25; xylan 7.13 ± 0.10; arabinan 0.79 ± 0.00; acetyl groups 0.15 ± 0.02; Klason lignin 10.02 ± 0.18; ethanol extractives 14.32 ± 1.01; ash 2.37 ± 0.05; uronic acids (as galacturonic acid equivalents) 3.73 ± 0.55, rest 6.76 (by difference).

These results were in agreement with the ones achieved by Araújo et al. [41], who reached a total carbohydrate content for avocado seeds of 64.61% (similar to the obtained in this work of 62.65%). However, this work reports a higher content than that obtained for other seeds such as argan kernels and black cumin oil seeds of 31.33 and 51.31 g/100 g of feedstock, respectively [42]. Additionally, similar ethanol extractives (10.54%) and carbohydrate content (50.20%) were obtained for olive stone but were found with higher lignin content (38.87%) [43]. Nonetheless, similar acid insoluble lignin content was obtained for other agricultural residue such as potato peel (15.94%) [44].

### 3.2. Selection of Solvent

In order to evaluate the benefits of using a deep eutectic solvent (DES) rather than an organic solvent, choline chloride:glycerol (1:1) and ethanol at 50% *v*/*v* and 80% *v*/*v* (that are typically used for polyphenols extraction) were employed. This DES was selected since it was already reported to extract phenolic compounds [18,45] and this molar ratio still maintains the key interactions among the compounds if compared with the typical molar ratio 1:2 [46]. Moreover, 30% water was included in the DES formulation to lessen the viscosity and ease the phenolic compounds solubilization [47].

**Figure 1:**
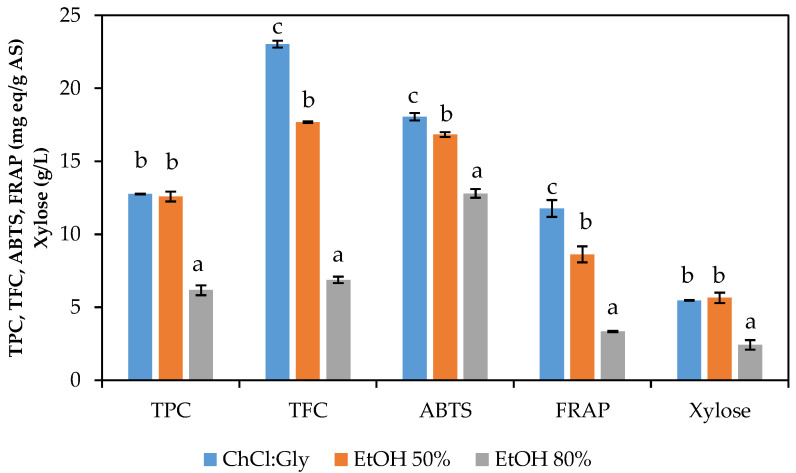
TPC (mg GAE/g avocado seed), TFC (mg RE/g avocado seed), ABTS, FRAP (mg TE/g avocado seed) and xylose content (g/L) of the liquors after extraction with choline chloride: glycerol (1:1) DES (30% water content), ethanol 50% (*v*/*v*) and ethanol 80% (*v*/*v*) at 50 °C for 2 h. Different letters imply different at a 95% confidence level.

Figure 1 displays the results obtained after the extraction of avocado seed with different solvents at 50 °C for 2 h. In general trends, both ethanol 50% and 80% achieved lower values of phenolic (TPC) and flavonoid content (TFC), antioxidant capacity (measured via ABTS and FRAP) and xylose content (no oligomers were identified) than when using the chlorine chloride:glycerol DES, finding statistical differences (*p* < 0.05) for TFC, ABTS, and FRAP among the three samples. Alternatively, TPC and xylose content were not significantly different for DES and ethanol 50% but were significantly different (at a 95% significance level) with the extracts from ethanol 80%.

In this sense, it was demonstrated that the DES employed enabled the recovery of an extract composed of phenols and flavonoids with higher antioxidant capacity and xylose content than that obtained from the ethanol extraction. Similar behavior was observed by other authors, obtaining higher TPC value when using DES than when using methanol, ethanol or water [45,48]. In the same context, Rodríguez-Martínez et al. [19] reported significant differences (*p* < 0.05) for the TPC and TFC values obtained for avocado peel extracted with choline chloride:glycerol (1:3 with 30% of water in its formulation) or with ethanol (96%), reaching 3.1 and 1.6-fold higher values when using the choline chloride:glycerol DES for TPC and TFC, respectively [19]. For that reason, that DES was selected as the solvent for the following experimental design.

### 3.3. Optimization of Extraction Conditions with DES

To obtain the extracts with the highest phenolic and flavonoid content, antioxidant capacity and xylose content, an experimental design based on response surface methodology (RSM) was applied. Table 1 displays the independent variables (temperature, time, and water content of the DES) and the results for dependent variables obtained (TPC, TFC, ABTS, FRAP, and xylose content). Table 2 exhibits the regression coefficients resulted for each model regarding a second-degree polynomial, the statistical significance (grounded on Student’s *t*-test), the parameters related to correlation (R^2^) and statistical significance (Fisher’s F test) of the models. The value obtained of R^2^ was close to 0.90 in all cases, which implies a good adequacy of the model to reflect the real relation among set variables. In addition, high values of F prove a good fitting of data. The *p*-value for each equation term was determined to evaluate the influence of linear interaction, and quadratic effects of the independent variables.

The determined significant regression coefficients, superior to 90% of confidential level, were employed to calculate five quadratic regression equations for the TPC (y_1_), TFC (y_2_), ABTS (y_3_), FRAP (y_4_) and xylose (y_5_):

(1) $$\mathrm{TPC}=14.76+1.18x_{2}+5.17x_{3}-2.42x_{1}^{1}$$

(2) $$\mathrm{TFC}=27.01+2.46x_{1}+5.75x_{3}-5.69x_{1}^{1}$$

(3) $$\mathrm{ABTS}=19.16+3.11x_{3}-3.31x_{1}^{1}-5.06x_{3}^{3}$$

(4) $$\mathrm{FRAP}=12.79+2.43x_{3}-3.31x_{1}^{1}-2.16x_{3}^{3}$$

(5) $$\mathrm{Xylose}=5.67+1.24x_{3}-1.27x_{3}^{3}$$

#### 3.3.1. Total Phenolic Content (TPC)

**Figure 2:**
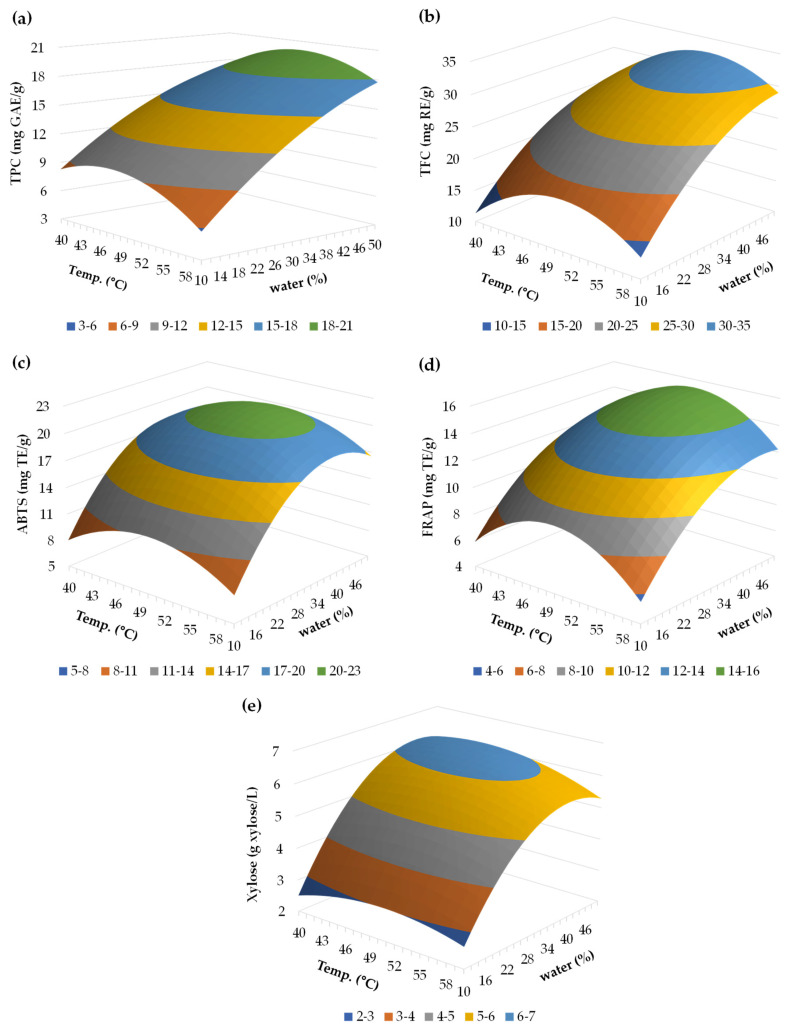
Response surface for (**a**) TPC (mg GAE/g AS), (**b**) TFC (mg RE/g AS), (**c**) ABTS (mg TE/g AS), (**d**) FRAP (mg TE/g initial avocado seed) and (**e**) xylose (g xylose/L), in function of time and amount of water. The variable set was time = 180 min.

The TPC values varied in the interval of 4.01–19.90 mg GAE/g initial AS (see Table 1), corresponding the minimum and maximum with experiments 7 and 13, respectively. In this sense, higher water content in the DES (50%) and milder conditions of temperature (50 °C) facilitate the recovery of phenolic compounds, in comparison to lower water content (10%), higher temperature (60 °C) and shorter residence times. This behavior can also be observed in the response surface of TPC displayed in Figure 2a, representing the interaction effect of temperature and water content setting the extraction time in 180 min (x_2_ = 1).

Regarding the regression coefficients (Table 2), both residence time and water content presented a significant effect on TPC (y_1_) value, as well as the quadratic effect of temperature. However, the combination of temperature with time or water content was not significant. In this sense, the plot reflects that the TPC was more affected by the water content than by the temperature, which is related to the high negative impact of the quadratic term of temperature on TPC equation. This behavior was also reported in other studies. For instance, Zannou and Koca [49] studied the effect of water and molar ratio in the extraction with sodium acetate:formic acid DES of alkanet for antioxidant phenolics, resulting in high water content were significantly beneficial for that aim. In addition, Alañón et al. [50], when extracting polyphenols from olive leaves, also reported that increasing the water content may facilitate phenolic recovery, although a certain amount should not be surpassed since the DES mixture may break down [51].

The results obtained in this work were in the range of those obtained by Permal et al. [52] for extruded avocado seed with 12.9 mg GAE/g. On the other hand, results of black mustard seed extracted with choline chloride:urea (1:2) DES, blended with 25% water, exhibited a TPC of 24.0 mg GAE/g under conditions of LSR = 10 mL/g, at 65 °C for 3 h [53]. Conversely, other seeds such as grape seeds reached higher TPC values of 56.17–156.17 mg GAE/g when extracted with choline choline:glucose (1:1) DES with 30% water content in an ultrasonic bath for 30 min at 50 °C [54].

#### 3.3.2. Total Flavonoid Content (TFC)

In this case, TFC was positively influenced by temperature (x_1_) and water content (x_3_), whereas the quadratic effect of temperature ($x_{1}^{1}$) reflected a negative effect. The results obtained ranged 7.98–32.10 mg RE/g AS, and the variation of TFC with temperature and water content (fixing the independent variable in a time of 180 min) can be observed in Figure 2b. Similar to what happened with TPC, a temperature of 50 °C and a high water content (50%) enabled the release of a higher amount of flavonoids. It must be highlighted that the water content presented a more intense impact than the temperature for the recovery of flavonoids (in fact, the temperature presented a large negative quadratic coefficient).

The results obtained can be positively compared to Weremfo et al. [55], who also reached similar values of TFC when subjecting avocado seed to microwave-assisted extraction with aqueous ethanol. In the same line, up to 37 mg RE/g were obtained when processing *Polygonum aviculare* leaves with choline chloride:glycerol (1:1) with 30% water content at 50 °C and 60 min in an ultrasonic bath at 80 W [56]. Additionally, Chen et al. [57] also found that the increasing of water content up to 30% in certain DES (for instance choline chloride:glycerol, 1:2) may increase the flavonoid content, reaching similar values to those obtained in this work when processing Rubia fruits powder in a sonication bath.

#### 3.3.3. Antioxidant Capacity (ABTS and FRAP)

The variation of antioxidant capacity of the extracts of avocado seed was evaluated by two antioxidant assays: ABTS and FRAP. In both cases, the values were positively affected by the water content, whereas the quadratic coefficient for temperature and water content was affected negatively (see Table 2).

Figure 2c exhibits the antioxidant capacity of the avocado seed extracts measured via ABTS assay with a set residence time of 180 min (x_2_ = 1). In this case, temperature of 50 °C and water content between 30–50% enabled reaching higher values of ABTS, with a maximum experimental value of 21.92 mg TE/g AS for the experiment 11 (temperature of 50 °C, time of 180 min, water content of 30%).

Figure 2d shows the impact of temperature and water content on the antioxidant capacity quantified by FRAP, with a set time of 180 min (x_2_ = 1). A similar behavior to ABTS is reflected in the FRAP plot, which value increased with a temperature of 50 °C and at larger amounts of water. The maximum value was 16.20 mg TE/g AS, obtained at 50 °C, 180 min and 30% water content (experiment 11). This value was in the range of avocado seed after extrusion with a value of 15.4 mg/g [52]. Conversely, higher values of FRAP were obtained from avocado peel extracted with choline chloride:glycerol (1:3) with 30% water content, reaching a value of 84.5 mg TE/g avocado peel after being processed for 120 min at 50 °C [19].

#### 3.3.4. Xylose Content

The results for xylose content also revealed that a temperature of 50 °C, larger residence time of 180 min, and 30% of water content assisted the recovery of extracts with a higher xylose content (see Figure 2e).

The xylose content was mainly and positively affected by the water content (x_3_) at a significant level, whereas the quadratic effect of water ($x_{3}^{3}$) had a meaningful negative impact (see Table 2) and the values ranged 2.08–6.05 g/L.

It is noteworthy that all the xylan obtained in the extract was in the form of monomeric xylose, rather than as xylooligosaccharides, which can be related to the fact that the DES may hydrolyze xylan into monomeric sugars, and also to the fact that the structure of xylan within the avocado seed may be arranged as a branch of other bigger polysaccharides (such as glucan), so the degree of polymerization of xylan in the raw avocado seed would be lower than for other feedstock. This fact is interesting since the solublization of xylan commonly occurs at higher temperatures of around 140–240 °C under hydrothermal treatment or using an acidified media [58].

### 3.4. Optimization of the Conditions and Validation of the Model

The goal of the experimental design was to evaluate the extraction conditions that may deliver the highest xylose content, and phenolic and flavonoid content with the highest antioxidant capacity measured via ABTS and FRAP. Statgraphics Centurion XVI software was employed to determine those conditions by converting the response values of each variable employing a desirability function, which was considered to reveal the combination of the extraction variables assessed able to maximize the set responses (TPC, TFC, ABTS, FRAP, and xylose) simultaneously.

In this context, the conditions leading to the greatest results of those dependent variables were considered as optimum: temperature of 50.10 °C, time of 180 min, and water content of 44.64%. Table 3 displays both the predicted and experimental values obtained at the selected optimum conditions. The applicability of the response surface approach for quantitative prediction was confirmed by the satisfactory agreement between predicted and experimental results. These values warranted the choice of the experimental design, which displayed high accuracy and reliability for the prediction of total phenolic content, flavonoids, antioxidant capacity, and xylose content of avocado seeds extracted with DES.

Under optimum conditions of extraction, the TPC value reached 19.71 mg GAE/g AS, TFC 33.41 mg RE/g AS, antioxidant capacity measured by ABTS 22.57 mg TE/g AS and by FRAP 16.64 mg TE/g AS and xylose content of 5.47 g/L. These results happened to be very similar to those found optimal when employing the single objective functions: 20.12 mg GAE/g AS, 32.89 mg RE/g AS, 21.43 mg TE/g AS, 15.59 mg TE/g AS, and 6.31 g xylose/L.

Regarding the spent solid after the optimized phenolics extraction, the solid yield was 79.82 g phenolic-free AS/100 g, and the chemical composition was (expressed as g of component/100 g of AS in dry weight ± standard deviation): glucan 68.26 ± 0.08, hemicelluloses 4.16 ± 0.45, lignin 12.16 ± 0.57. The glucan and lignin content corresponded to a practical quantitative recovery of these two fractions within the solid matrix. A similar performance was observed by Del Castillo-Llamosas et al. [59] when processing avocado peel via non-isothermal autohydrolysis at 140–180 °C, recovering around 90% of initial glucan and lignin. The only fraction affected was xylan from the hemicellulosic fraction, as was already explained in previous sections.

In this sense, in order to apply a biorefinery scheme based on the manufacture of multiple products, the extraction of antioxidants phenolics may be combined with the employment of the spent solid with other purposes, for instance, the manufacture of glucose that could be employed for the production of ethanol, succinic acid or lactic acid, inter alia, which will be explored in following sections.

### 3.5. Identification of Phytochemicals

Table 4 displays the phytochemical profile, identified by HPLC-ESI, from the liquid fraction (DES) obtained at optimal conditions. Among the identified phytochemicals there are six phenolic acids and two flavonoids. The chromatograms of the phytochemicals identified by HPLC-ESI can be consulted in Figure S1.

Some of these compounds were also identified in other extracts from avocado seed. Saavedra et al. [60] studied the convective drying process as a way to preserve functional compounds, observing 1.17 mg ferulic acid/100 g of seed. Similarly, Pahua-Ramos et al. [61] subjected avocado seed to Soxhlet extraction with methanol (75%), identifying 128.18 µg of protocatechuic acid and 28.67 µg of vanillic acid per g of seed.

On the other hand, those compounds were observed as well in extracts coming from avocado peel. Ferulic acid (up to 8 mg/100 g) was also found by Rodríguez-Martínez et al. [19] in the extract obtained by DES extraction from avocado peel. Similarly, *p*-coumaric acid, ferulic acid, vanillic acid, protocatechuic acid, and naringenin were also discovered in the extract of avocado peel subjected to autohydrolysis [59]. On the other hand, 128 mg apigenin/g of phenolic compound and 2.24 mg *p*-coumaric acid/100 g dry matter were observed in avocado peel extract by [62]. Saavedra et al. [60] obtained up to 6.21 and 1.9 mg of ferulic acid and naringenin per 100 g of avocado peel, respectively. Additionally, Figueroa et al. [5] employed a drying process and a pressurized liquid extraction on avocado peel, obtaining 11.4 mg protocatechuic acid/100 g, 7 mg vanillic acid/100 g, and 1.2 mag naringenin/100 g when drying at 45 °C. Moreover, naringenin (3.27–25.42 mg/100 g) and *p*-coumaric acid (0.72–5.21 mg/100 g) were also identified in avocado fruit as stated by Alkaltham et al. [63].

### 3.6. Processing of the Phenolic-Free Avocado Seed to Increase Enzymatic Digestibility: DES-Delignification or Microwave-Asssisted Autohydrolysis

With the final aim of increasing the enzymatic digestibility of avocado seed, two pretreatment strategies were carried out using the phenolic-free avocado seed (extracted under optimized conditions) as feedstock: (i) delignification using the same DES employed for phenolics extraction, and (ii) microwave-assisted autohydrolysis using water to solubilize the glucan into the liquid fraction.

For the first strategy, the solid yield was 92.94 g delignified AS/100 g, indicating that the process may have not been enough to solubilize the lignin fraction. The chemical composition of the spent solid comprised (expressed as g of component/100 g of AS in dry weight ± standard deviation): glucan 65.53 ± 0.39, hemicelluloses 4.52 ± 0.10, lignin 11.82 ± 0.37. In this sense, 88% of glucan (regarding glucan from initial AS) remained in the solid fraction, whereas 13% of the lignin was solubilized into the DES. ChCl:glycerol (1:2) was employed by Lee et al. [64] on oil palm empty fruit bunch using ultrasound-assisted pretreatment under selected conditions (50 °C, 53 kHz, 210 W, 30 min) to obtain a delignification of around 10%. The mixture choline chloride, glycerol, and iron (III) chloride applied on corn stover at 120 °C for 4 h enabled the solubilization of up to 48% of the lignin, producing the solubilization of almost 16% of the glucan [65].

Alternatively, after the microwave-assisted autohydrolysis, the solid yield achieved was 31.29 g autohydrolyzed AS/100 g. The chemical composition of the spent solid was (expressed as g of component/100 g of AS in dry weight ± standard deviation): glucan 17.82 ± 0.50, hemicelluloses 2.51 ± 0.27, lignin 12.89 ± 1.13. Additionally, the liquid fraction was mainly composed of oligomers (expressed in g/L): glucooligosaccharides 51.09 ± 0.09, xylooligosaccharides 2.43 ± 0.01, acetyl groups linked to oligosaccharides 0.11 ± 0.02 and small amounts of monomers and acetic acid (expressed in g/L): glucose 0.18, xylose 0.20, arabinose 0.19, and acetic acid 0.11). In this sense, the solubilization of glucan was practically total (in the form of glucooligosaccharides), only remaining in the solid fraction at about an 8% regarding initial glucan content. Similarly, Araújo et al. [66] reached higher starch extraction yields when operating at temperatures higher than 180 °C using microwave-assisted extraction with water over avocado seed.

### 3.7. Enzymatic Hydrolysis of the Spent Solids of Avocado Seed after Processing

**Figure 3:**
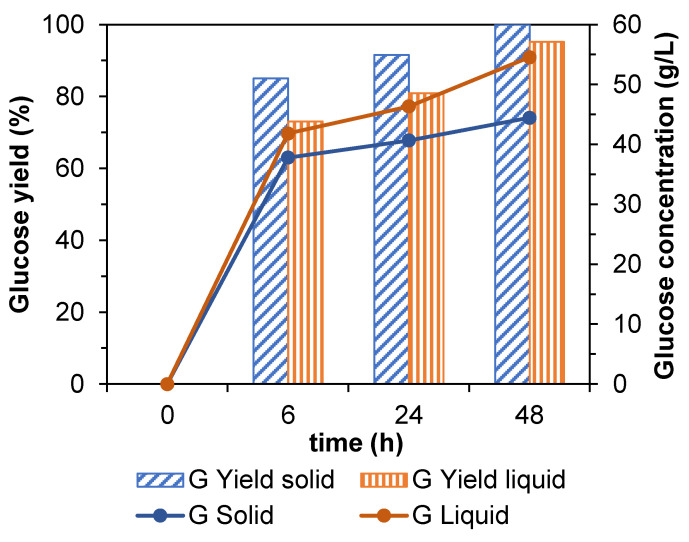
Glucose yield (%) and concentration (g/L) during the enzymatic hydrolysis of the DES pretreated spent solid (blue) and the liquor from microwave-assisted autohydrolysis (orange). Standard deviation is lower than 1%.

In order to estimate the influence of the pretreatments over phenolic-free avocado seed to increase enzymatic digestibility, the solid phase after delignification (LSR = 20 g/g) and liquid phase after microwave-assisted autohydrolysis, were used as substrates in enzymatic hydrolysis tests under fixed conditions (ESR = 25 FPU/g and β-glucosidase/cellulase ratio = 5 IU/FPU for both cases). The concentration (g/L) and glucose yield (%) data obtained in these two sets of experiments at 6, 24, and 48 h are represented in Figure 3. In the case of the solid from DES-delignification (blue color) after a saccharification time of 48 h, the glucose concentration was around 45 g/L and the yield reached 100%. These data, compared positively to enzymatic digestibility of phenolic-free AS solid (data not included in the current study), showing an improvement of more than 50%, thus concluding that pretreatment with DES is an appropriate method since it allows the increase in the digestibility of the raw material in addition to obtaining a separated stream rich in lignin. Additionally, it is known that DES can efficiently remove lignin and hemicelluloses from lignocelluloses materials but keep the glucan structure intact [67,68]. Betwixt the main advantages of using DES is that they are affordable, recyclable, biodegradable, biocompatible, and produce fewer toxic by-products [69,70]. Other studies carried out for different raw materials reported improvements in the digestibility of the solid after the application of DES in the pretreatment stage. In the study conducted by Yan et al. [71], corn cob was pretreated with a DES consisting of choline chloride and oxalic acid (1:1), achieving a digestibility close to 70%. Additionally, the research carried out by Lynam et al. [72] in loblolly pine concluded that, using a mixture of formic acid and choline chloride, the yield in glucose increased by seven times. On the other hand, Zhang et al. [73] predated corncob with a DES conformed for lactic acid and choline chloride (10:1), achieving a glucose yield of 80% after enzymatic hydrolysis.

For the liquid from microwave processing (orange color in Figure 3), it was observed that a glucose concentration of 55 g/L and a yield of 95% were obtained in 48 h. As in the previous pretreatment, this pretreatment improves significantly the enzymatic digestibility (almost 55% for the glucose concentration and yield) if compared to the results obtained with the phenolic-free AS solid. This pretreatment was also employed by other authors. For example, microwave-assisted autohydrolysis that was employed on Paulownia wood (230 °C 0.5 min) reached a glucan to glucose conversion close to 80% at 72 h [74]. Additionally, Amini et al. [75] reached almost quantitative conversion after enzymatic hydrolysis of eucalyptus sawdust subjected to hot water treatment assisted with microwaves.

### 3.8. Mass Balance of the Overall Process

**Figure 4:**
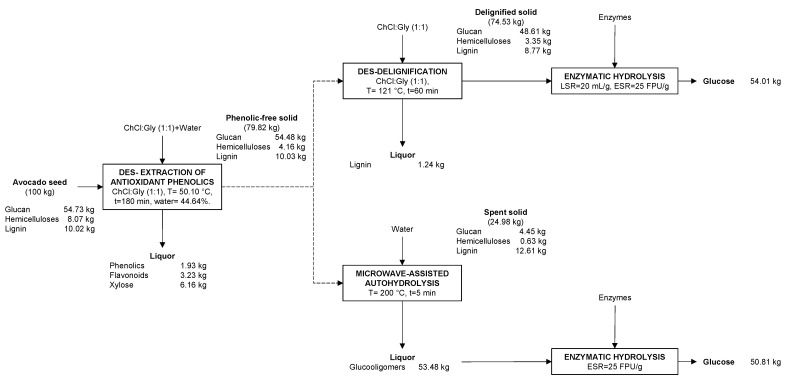
Overall mass balance of the process, considering kg/100 kg of AS (on dry basis).

With the final aim of achieving a comprehensive knowledge of the processes carried out within this work, a mass balance is displayed in Figure 4. Firstly, 100 kg of dry AS would be subjected to extraction of antioxidant phenolics, achieving 1.93, 3.23 and 6.16 kg of phenolics, flavonoids, and xylose, respectively, in the liquor. After that extraction, 79.82 kg of phenolic-free solid were obtained and would be subjected to two different strategies: (i) delignification with DES, and (ii) microwave-assisted autohydrolysis.

For the first strategy, ChCl:glycerol was employed at 121 °C for 1 h, enabling the solubilization of 1.24 kg of lignin. On the other hand, the delignified solid comprised mainly glucan (48.61 kg) that would be transformed into 54.01 kg of glucose (glucan to glucose conversion of 100%).

For the second strategy, the phenolic-free solid was mixed with water and subjected to microwave-assisted autohydrolysis at 200 °C for 5 min. The spent solid (24.98 kg) was mainly composed of lignin (12.61 kg) and remains of glucan (4.45 kg) and hemicelluloses (0.63 kg). Regarding the liquid fraction, it was mainly composed of glucooligosaccharides (53.48 kg) that were subsequently transformed into 50.81 kg of glucose after enzymatic hydrolysis (glucan to glucose conversion of 95%).

In this sense, both strategies would enable to recover, on the one hand, antioxidant phenolics and xylose in a first extraction stage, and a substrate (either solid for DES-delignification or liquid for microwave-assisted autohydrolysis) with high enzymatic digestibility, that may allow for obtaining other valuable compounds, such as bioethanol. If all the glucose were transformed into ethanol, between 25.97–27.61 kg of ethanol could be obtained from 100 kg of AS. This can be positively compared to other raw materials, for instance, corn stover subjected to microwave-assisted autohydrolysis (200 °C 30 min) would reach around 15.93 kg of ethanol per 100 of corn stover [76]. In addition, a similar amount of ethanol (20.66 kg) could be obtained from potato peel waste treated by liquefaction [77].

## 4. Conclusions

The optimization of antioxidant phenolics and xylose was efficaciously performed using avocado seed residue and deep eutectic solvents. The data obtained from the experimental designed demonstrated that the water content and the quadratic effect of temperature and water produced the highest impact on the parameters measured (TPC, TFC, ABTS; FRAP, and xylose content). The optimized extraction conditions were 50.10 °C for 180 min with a water content of 44.64%, obtaining coherent values for the predicted and experimental values and identifying eight phenolic compounds by HPLC-ESI. As a second stage of this biorefinery, DES-delignification and microwave-assisted autohydrolysis were carried out to increase the enzymatic digestibility of the remain glucan, reaching a glucose conversion of 95–100% for both cases. This strategy demonstrated the effective valorization of avocado seed in two stages, enabling the recovery of antioxidant phenolics, xylose and highly enzymatically digestible glucan/glucooligosaccharides, hence leading from a waste to several products of value.

## Figures and Tables

**Table 1:** Operational conditions (expressed as dimensionless or dimensional (in brackets) independent variables) evaluated, and results reached for dependent variables y_1_ to y_6_.

Exp.	X_1_-T (°C)	x_2_-t (min)	x_3_-Water (%)	y_1_-TPC	y_2_-TFC	y_3_-ABTS	Y_4_-FRAP	Y_5_-Xylose
**1**	1 (60)	1 (180)	1 (50)	18.02	30.18	17.22	12.12	5.64
**2**	1 (60)	1 (180)	−1 (10)	4.59	10.40	6.64	4.50	2.08
**3**	1 (60)	−1 (60)	1 (50)	15.00	23.40	13.54	10.10	4.71
**4**	1 (60)	−1 (60)	−1 (10)	5.51	17.50	11.60	7.75	4.24
**5**	−1 (40)	1 (180)	1 (50)	16.82	22.01	14.65	11.00	5.53
**6**	−1 (40)	−1 (60)	1 (50)	14.07	19.30	12.41	8.70	5.21
**7**	−1 (40)	−1 (60)	−1 (10)	4.01	7.98	6.29	4.60	1.93
**8**	−1 (40)	1 (180)	−1 (10)	9.45	12.30	8.91	6.20	2.98
**9**	1 (60)	0 (120)	0 (30)	14.70	25.70	17.72	11.40	5.71
**10**	−1 (40)	0 (120)	0 (30)	11.70	21.00	15.64	9.10	4.93
**11**	0 (50)	1 (180)	0 (30)	16.56	32.10	21.92	16.20	6.05
**12**	0 (50)	−1 (60)	0 (30)	15.08	27.00	19.70	12.90	5.64
**13**	0 (50)	0 (120)	1 (50)	19.90	31.00	18.28	14.10	5.73
**14**	0 (50)	0 (120)	−1 (10)	8.53	20.20	11.58	8.70	3.22
**15**	0 (50)	0 (120)	0 (30)	14.28	24.10	18.00	12.00	5.57
**16**	0 (50)	0 (120)	0 (30)	13.60	23.60	18.40	11.60	5.62
**17**	0 (50)	0 (120)	0 (30)	13.00	25.20	17.74	11.70	5.48

TPC, TFC, ABTS, and FRAP measured in mg equivalent/g of initial avocado seed, and xylose measured in g/L. Standard deviation was lower than 5%.

**Table 2:** Regression coefficients and statistical parameters measuring the correlation and significance of the models.

Coefficient	y_1_-TPC	y_2_-TFC	y_3_-ABTS	Y_4_-FRAP	Y_5_-Xylose
**x_0_**	14.76 ^a^	27.01 ^a^	19.16 ^a^	12.79 ^a^	5.67 ^a^
**x_1_**	0.18	2.46 ^b^	0.88	0.63	0.18
**x_2_**	1.18 ^c^	1.18	0.58	0.60	0.06
**x_3_**	5.17 ^a^	5.75 ^a^	3.11 ^a^	2.43 ^a^	1.24 ^a^
**x_12_**	−0.76	−0.92	−0.77	−0.64	−0.32
**x_13_**	0.69	0.58	0.08	0.13	−0.23
**x_23_**	0.16	1.53	1.03	0.75	0.30
**x_11_**	−2.42 ^c^	−5.69 ^b^	−3.31 ^b^	−3.31 ^a^	−0.43
**x_22_**	0.20	0.51	0.82	0.99	0.09
**x_33_**	−1.40	−3.44	−5.06 ^a^	−2.16 ^b^	−1.27 ^a^
**R^2^**	0.942	0.889	0.942	0.918	0.928
**F-exp**	12.71	6.21	12.61	8.75	9.95
**Significance level (%)**	99.85	98.75	99.85	99.54	99.69

^a^ Significant coefficients at the 99% confidence level. ^b^ Significant coefficients at the 95% confidence level. ^c^ Significant coefficients at the 90% confidence level.

**Table 3:** Predicted and experimental values under optimum conditions based on multiple response of TPC, TFC, ABTS, FRAP, and xylose (T = 50.10 °C, t = 180 min, water content = 44.64%).

	y_1_-TPC	y_2_-TFC	y_3_-ABTS	y_4_-FRAP	y_5_-Xylose
**Predicted value**	19.32	32.31	20.91	15.59	6.24
**Experimental value ^1^**	19.71 ± 0.28	33.41 ± 1.76	22.57 ± 0.61	16.64 ± 0.15	5.47 ± 0.03

^1^ Mean value (*n* = 3) ± standard deviation (SD) from three extraction replicates.

**Table 4:** Identification and measurement of the main phytochemicals from the optimal extraction of AS with DES. Standard deviation was lower than 5%.

Compound	Type	Content (mg/g Initial AS)
*p*-coumaric acid	Phenolic acid	0.43
Salicylic acid	Phenolic acid	0.27
Ferulic acid	Phenolic acid	0.15
Phthalic acid	Phenolic acid	0.85
Protocatechuic acid	Phenolic acid	0.39
Vanillic acid	Phenolic acid	0.52
Naringenin	Flavonoid	1.44
Apigenin	Flavonoid	0.13

## Data Availability

Data is contained within the article.

## Supplementary Materials

The following supporting information can be downloaded at: https://www.mdpi.com/article/10.3390/antiox12061156/s1, Figure S1: Chromatograms of the phytochemicals identified by HPLC-ESI.
