# Supplementary material for: Valorization of Avocado Seed Wastes for Antioxidant Phenolics and Carbohydrates Recovery Using Deep Eutectic Solvents (DES)
Source: Antioxidants (Basel). 2023 May 26;12(6):1156. doi: 10.3390/antiox12061156 (PMC10295328; doi:10.3390/antiox12061156)

Article

Valorization of avocado seed wastes for antioxidant phenolics and carbohydrates recovery using deep eutectic solvents (DES)

Alexandra Del-Castillo-Llamosas[1], Fernando Rodríguez-Rebelo[1], Beatriz Rodríguez-Martínez[1], Adrián Mallo-Fraga[1], Pablo G. Del-Río [1,2,*], Beatriz Gullón[1]

1. Universidade de Vigo, Departamento de Enxeñaría Química, Facultade de Ciencias, 32004 Ourense, Spain; alexandra.castillo@uvigo.es (A.C.D.L.), frodriguez@uvigo.es (F.R.R.), beatriz.rodriguez@uvigo.es (B.R.M.), pdelrio@uvigo.es (P.G.D.R.), bgullon@uvigo.es (B.G.).
2. Stokes Laboratories, School of Engineering, Bernal Institute, University of Limerick, Limerick, V94 T9PX, Ireland; pablo.garciadelrio@ul.ie (P.G.D.R.).

* Correspondence: pdelrio@uvigo.es (P.G.D.R.)

Fig S1. Chromatograms of the phytochemicals identified by HPLC-ESI.

p-coumaric acid

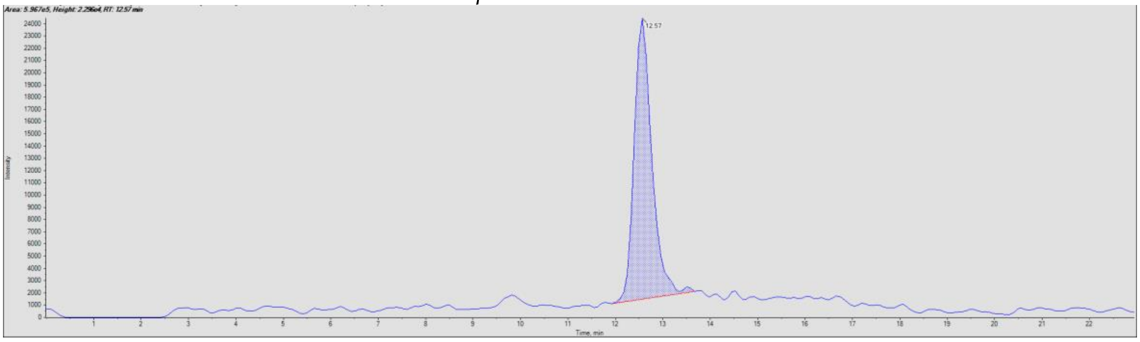

Salicylic acid

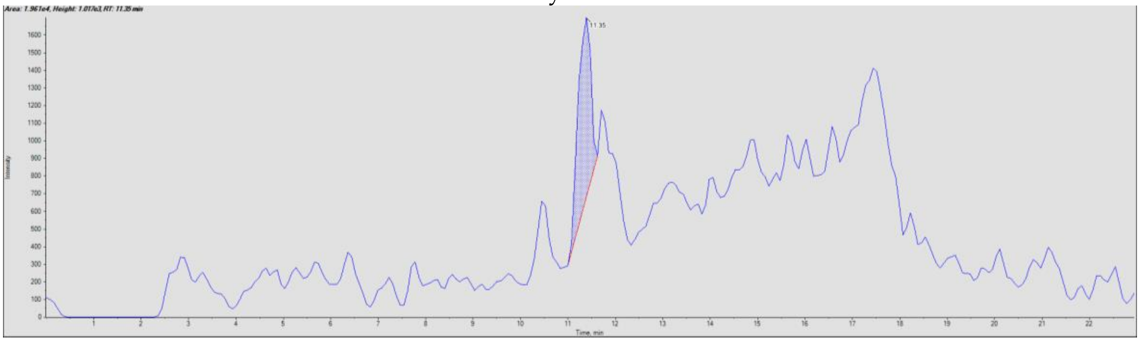

Ferulic acid

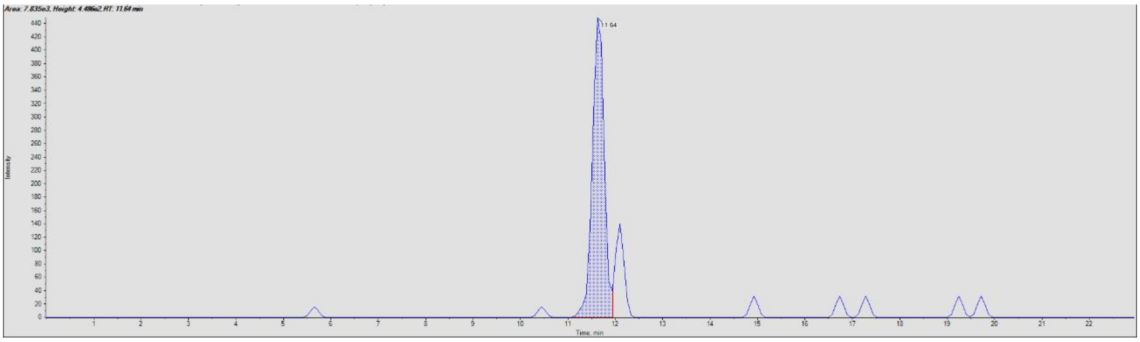

Phtalic acid

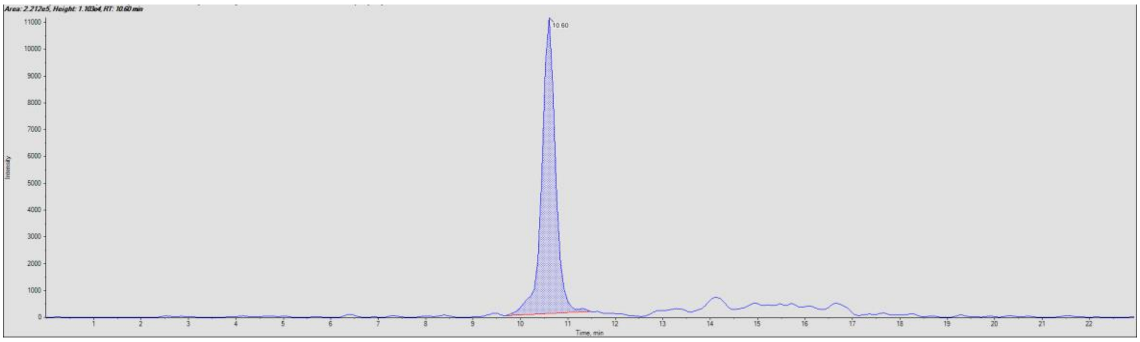

Protochatechuic acid

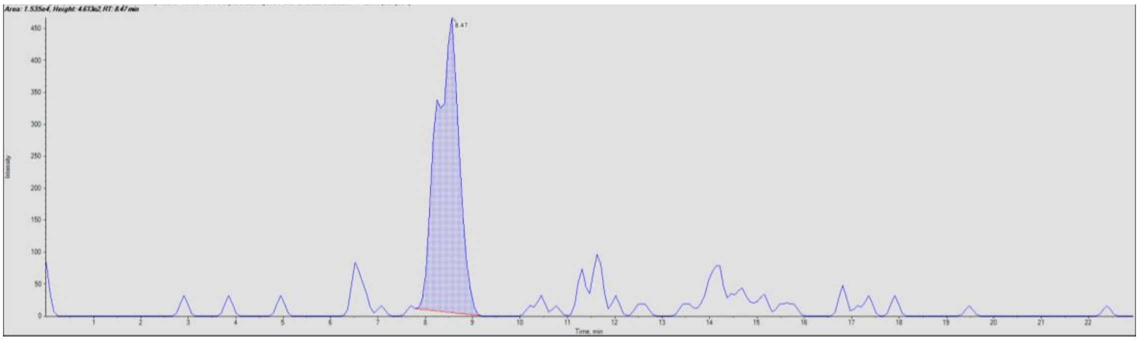

Vanillic acid

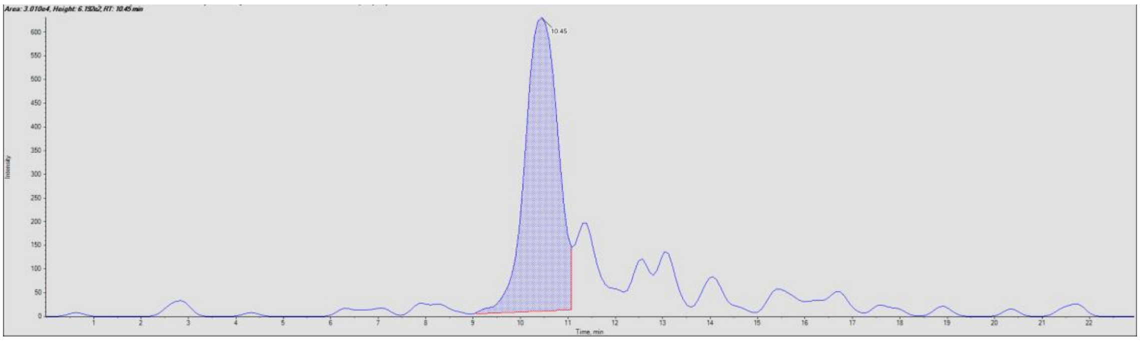

Naringenin

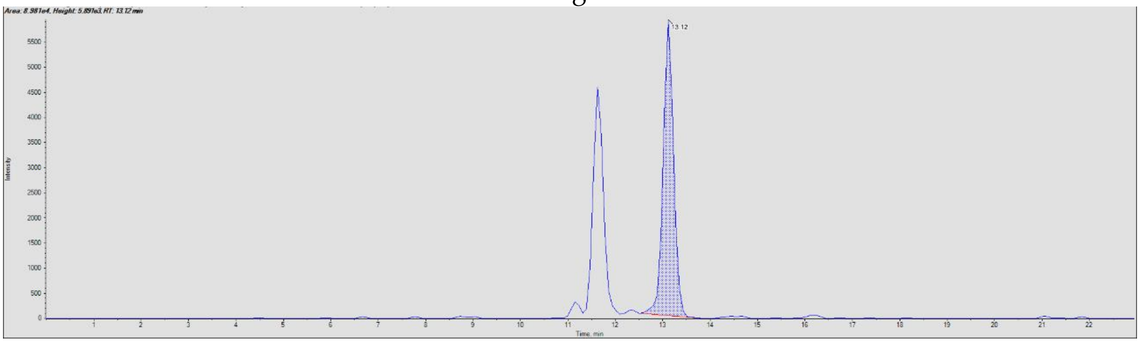

Apigenin

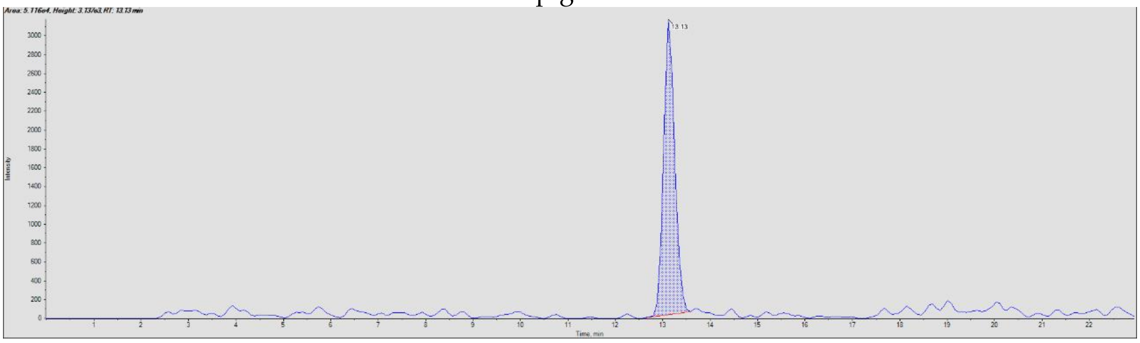

Supplement: Supplementary file 1 [file antioxidants-12-01156-s001.zip › antioxidants-2348547-supplementary.pdf]
